# Design and optimization of high temperature optocouplers as galvanic isolation

**DOI:** 10.1038/s41598-021-04145-3

**Published:** 2022-02-09

**Authors:** Abbas Sabbar, Syam Madhusoodhanan, Huong Tran, Binzhong Dong, Jiangbo Wang, Alan Mantooth, Shui-Qing Yu, Zhong Chen

**Affiliations:** 1 Department of Electrical Engineering, University of Arkansas, Fayetteville, AR 72701 USA; 2 HC SemiTek (Suzhou), 28 Chenfeng Road, Zhangjiagang, Suzhou, 215600 Jiangsu People’s Republic of China

**Keywords:** Engineering, Materials science, Physics

## Abstract

The commercial InGaN-based (blue and green) and AlGaInP-based (red) multiple quantum well (MQW) lighting emitting diodes (LEDs) were studied in a wide range of temperatures up to 800 K for their light emission and detection (i.e., LEDs operated under reverse bias as photodiodes (PDs)) characteristics. The results indicate the feasibility of integrating a pair of selected LEDs to fabricate high temperature (HT) optocouplers, which can be utilized as galvanic isolation to replace the bulky isolation transforms in the high-density power modules. A detailed study on LEDs and PDs were performed. The external quantum efficiency (EQE) of the LED and PDs were calculated. Higher relative external quantum efficiency (EQE) and lower efficiency droops with temperatures are obtained from the blue and green LEDs for display compared with the blue one for lighting and red LED for display. The blue for lighting and red for display devices show superior responsivity, specific detectivity (D*), and EQE compared with blue and green for display when operated as PDs. The results suggest that red LED devices for display can be used to optimize HT optocouplers due to the highest wavelength overlapping compared with others.

## Introduction

Power electronic systems are widely used in numerous applications, such as geothermal, gas turbines, combustion engines, and space explorations, to sustain the high-temperature (HT) ambient environments^1–4^. Some other applications (e.g., aerospace, heavy equipment, marine systems, and automotive), which are not necessarily HT ambient situations, also drive the demands for high-density power electronics with the requirements of low volume, lightweight, and high efficiency^5–10^. High-density power modules (HDPMs), the core component of power electronic systems, are expected to be operated at high temperatures to meet these stringent requirements. Wide bandgap (WBG) materials such as silicon carbide (SiC) and gallium nitride (GaN) are employed in HDPMs to overcome the temperature, frequency, and power density limitations of conventional Si-based power modules^10–13^. The SiC power module has been demonstrated with operating temperatures up to 525 K^14–16^.

Even though the high-temperature operation of the SiC power module is achievable, the temperature capability of the gate driver, isolation, sensing, and other passive components surrounding the power modules limits the development of HDPM. In the design of HDPM, the galvanic isolation on the gate driver board is required to pass through the gate control signal, reject the transient noise, and break the ground loops^17,18^. The current HT SiC power modules utilize large gate transforms to deliver galvanic isolation between high voltage power devices and low voltage gate drivers. The scaling of HDPMs is limited by the volume and heaviness of these gate transformers. Therefore, an optocoupler (i.e., lighting emitting diode (LED) and photodetector (PD)) with high-temperature operation is highly desirable as an alternative to the gate transformers. The HT optocouplers could lead to ultra-high-density 3-D power modules capable of achieving excellent thermal management, power density, power efficiency, reliability, and operating environment. Therefore, optocouplers (LEDs and PDs) that can consistently function up to 525 K are extremely attractive for next-generation high-density power electronics applications.

The development of HT optocouplers comprises an orderly approach, starting from evaluating high-temperature characteristics of LED materials and devices and utilizing the LEDs as detectors to construct optocoupler packaging. We first reported a systematic study of the spontaneous emission quantum efficiency (QE) of green LED materials up to 800 K^19^. Then, various commercial LED materials for lighting and display applications have been characterized and evaluated using the temperature-dependent power-dependent photoluminescence (PL) method^20^. Furthermore, developments in the LED quantum well (QW) structures were made for HT applications^21^. The study shows the impact of the QW structure layers on the spontaneous emission QE of commercial LED materials. The peak spontaneous emission QE of InGaN- based LED materials are (79–84)% and (48–60)% at 500 K and 800 K, respectively. After that, HT optical studies of blue LED for lighting (BL), blue LED for display (BD), and green LED for display (GD) are conducted for possible integration as an optocoupler emitter in HDPMs^22,23^. The internal quantum efficiency (IQE) is calculated using temperature and intensity-dependent electroluminescence (EL) measurements. All three LEDs exhibit more than 70% IQE at 500 K. At 800 K, IQE is above 40% for BD and GD LEDs. However, BL LED exhibits only 24% of IQE at 800 K. Also, an initial study on using GD LED as a PD is performed. The device shows a spectral response of 0.027 and 0.017 A/W at 500 K and 800 K, respectively. The signal to noise ratio, specific detectivity, D* is calculated as well. When the temperature increases from 77 to 800 K, the D* is decreased from 1 × 10^10^ to 4 × 10^8^ cmHz^1/2^ W^−1^. In this paper, we report on the selection of LEDs for HT optocouplers (i.e., one is for illumination, and the other is for light detection) since the LED can function either as a light source in forward biasing mode or as a PD in photovoltaic mode (unbiased) or reverse biased conditions. The work aims to fabricate HT optocouplers using different commercial blue, green, and red LEDs for lighting and display applications.

## Results

### LEDs characterizations

**Figure 1:**
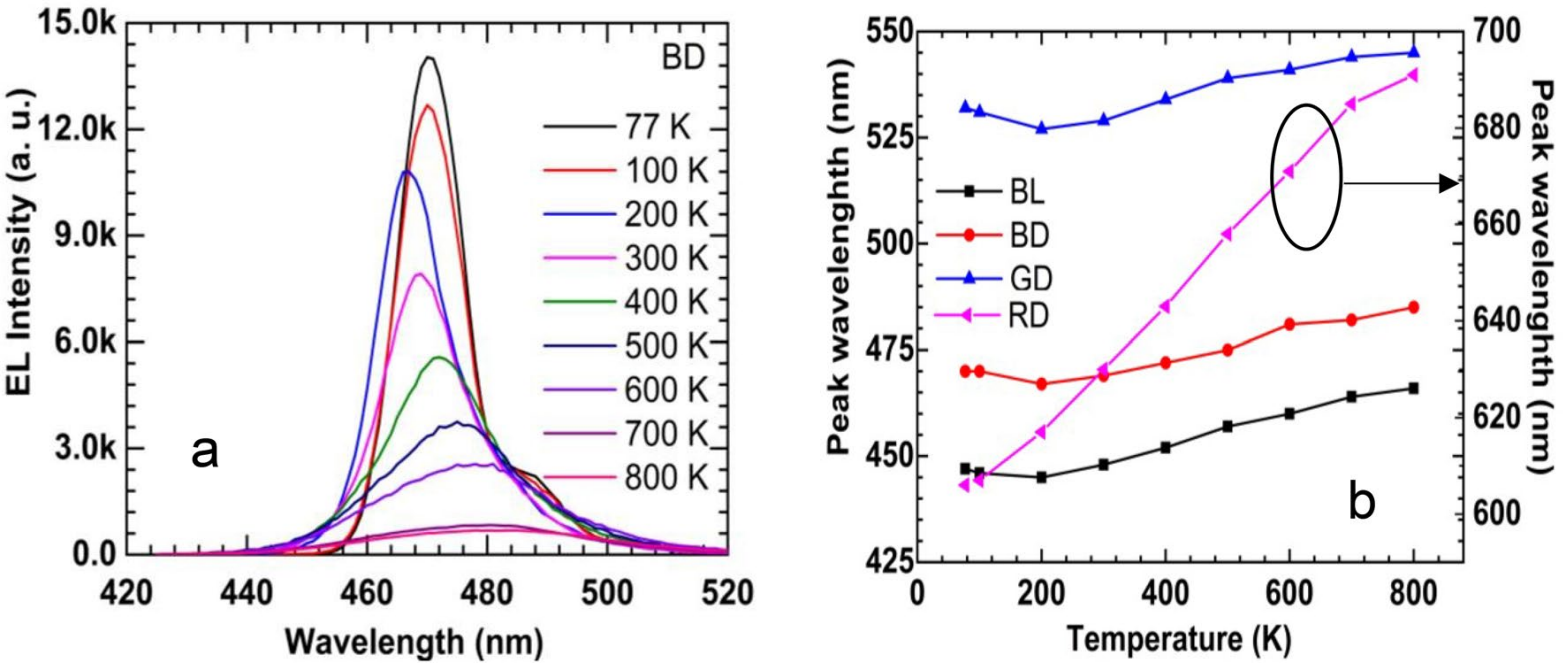
(**a**) Temperature-dependence EL spectra of blue for display under current injection of 0.3 mA. (**b**) Peak positions of EL spectra as a function of temperature for all devices.

Figure 1a shows a typical temperature-dependent EL spectra of the BD LED in temperature ranging from 77 to 800 K with an injection current of 0.3 mA to avoid the self-heating of the LEDs with a turn-on voltage of under 2.0 V^22,23^. The tail of the EL spectra on the high-energy region (i.e., lower wavelength) became broader as the temperature was increased due to the increasing number of the electron–hole pairs in the conduction and valance bands at elevated temperatures. Although the EL spectra decrease significantly at high temperatures, a clear EL signal is observed up to 800 K. Other LEDs (BL, GD, and RD) show similar behaviours in terms of dropping in the EL intensity and boarding the EL spectra as the temperature is increased. It is observed the peak wavelength is temperature-dependent as well, and it is varied depending on the device type, as shown in Fig. 1b. The temperature-dependence peak wavelength of LEDs influences the paring of LEDs and PDs. The InGaN-based LEDs show less temperature-dependent peak wavelength than the AlGaInP-based LEDs. Samples BL, BD, and GD and exhibit blueshifts at low temperatures. A redshift is observed above 300 K. On the other hand, only a redshift is observed in sample RD. As the temperature is increased from 77 to 800 K, the peak wavelength changes by 19, 15, 13, and 85 nm for samples BL, BD, GD, and RD, respectively.

**Figure 2:**
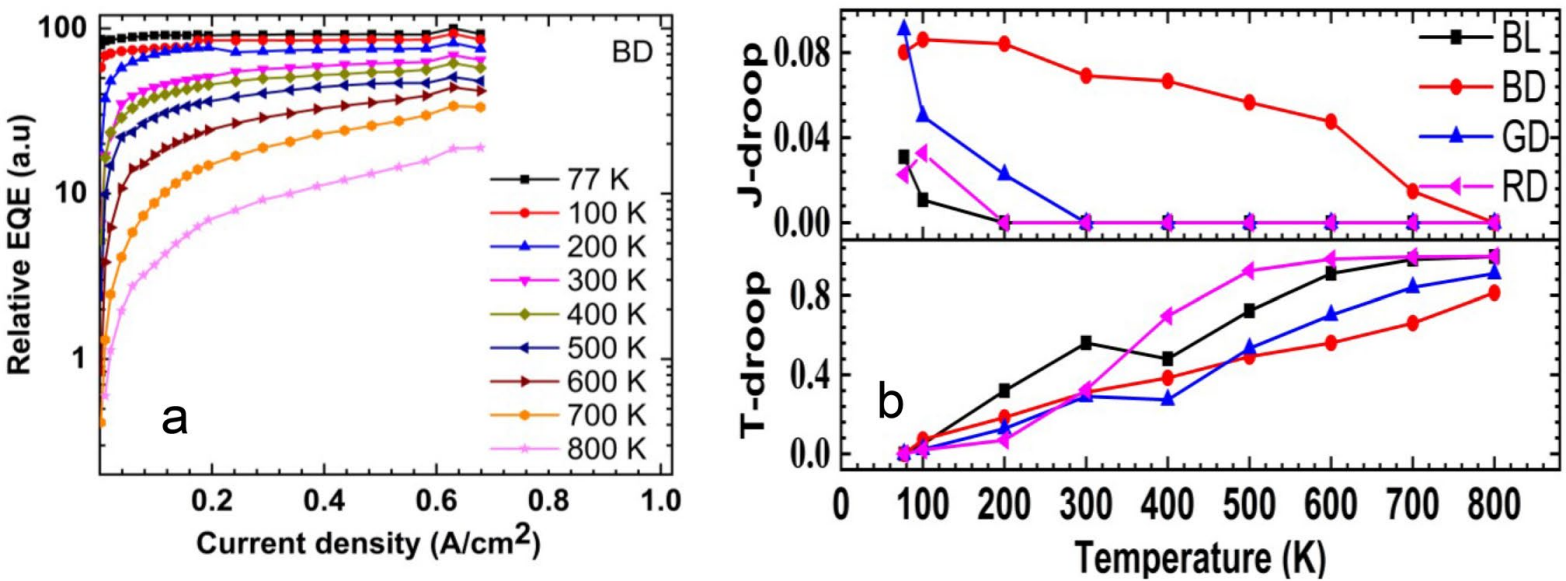
(**a**) Temperature-dependence relative EQE versus current density of blue for display. (**b**) Temperature dependence J-droop and T-drop of blue for lighting and display, green for display, and red for display.

Figure 2a shows temperature-dependent EQE for sample BD. The EQE is normalized by the peak EQE at 77 K. The EQE is increased by increasing the current density, and it is decreased by increasing the temperature. A similar trend is observed for the other LED devices. The relative EQE of sample B is reduced by 80 % by increasing the temperature from 77 to 800 K.

The current-dependent efficiency droop, the so-called “J-droop,” and the temperature-dependent efficiency droop, often referred to as the “T-droop.” Both the J-droop and T-droop are essential factors determining the performance of LEDs, so there is a need to investigate and compare the InGaN-based blue for light and display and green for display LEDs and the AlGaInP based red LED. The J-droop and the T-droop can be calculated as follow^24^:

1 $$J{\text{-}}droop = \frac{{Peak\;EQE{\text{--}}EQE\;at\;\max .\;current\;density}}{{Peak\;EQE}}$$

2 $$T{\text{-}}droop = \frac{{Peak\;EQE\;at\;77\;K - peak\;EQE\;at\;T}}{{Peak\;EQE\;at\;77\;K}}$$

The InGaN based BL, BD, and GD LEDs and the AlGaInP based RD LED are compared. Figure 2b shows the efficiency droop in the LEDs, calculated from the EQE^24^. The J-droops of the BL, BD, GD, and RD are 0.03, 0.08, 0.09, and 0.02 at 77 K. They all decrease to 0 at 800 K, indicating that current density is not enough to play a significant role in the efficiency drop of the LEDs. The maximum current density used was 0.27 A cm^−2^ for BL LED, 0.67 A cm^−2^ for BD and GD LEDs, and 3.3 A cm^−2^ for RD LED. Typically, the J-droop is more pronounced at high current density, which was intentionally avoided in our measurements to prevent any damage to the LEDs at HTs^22^. On the other hand, the T-droop shows an opposite behaviour to the J-droop. All samples are not shown an efficiency droop at 77 K. However, the T-droop becomes more pronounced as the temperature increases. The T-drop of the BL, BD, GD, and RD is 0.99, 0.81, 0.90, and 0.99 at 800 K, which implies the T-droop plays a significant role in the efficiency drop of the LEDs at HTs. Moreover, the influence of the T-droop is more remarkable on the BL and RD LEDs.

### PDs characterization

**Figure 3:**
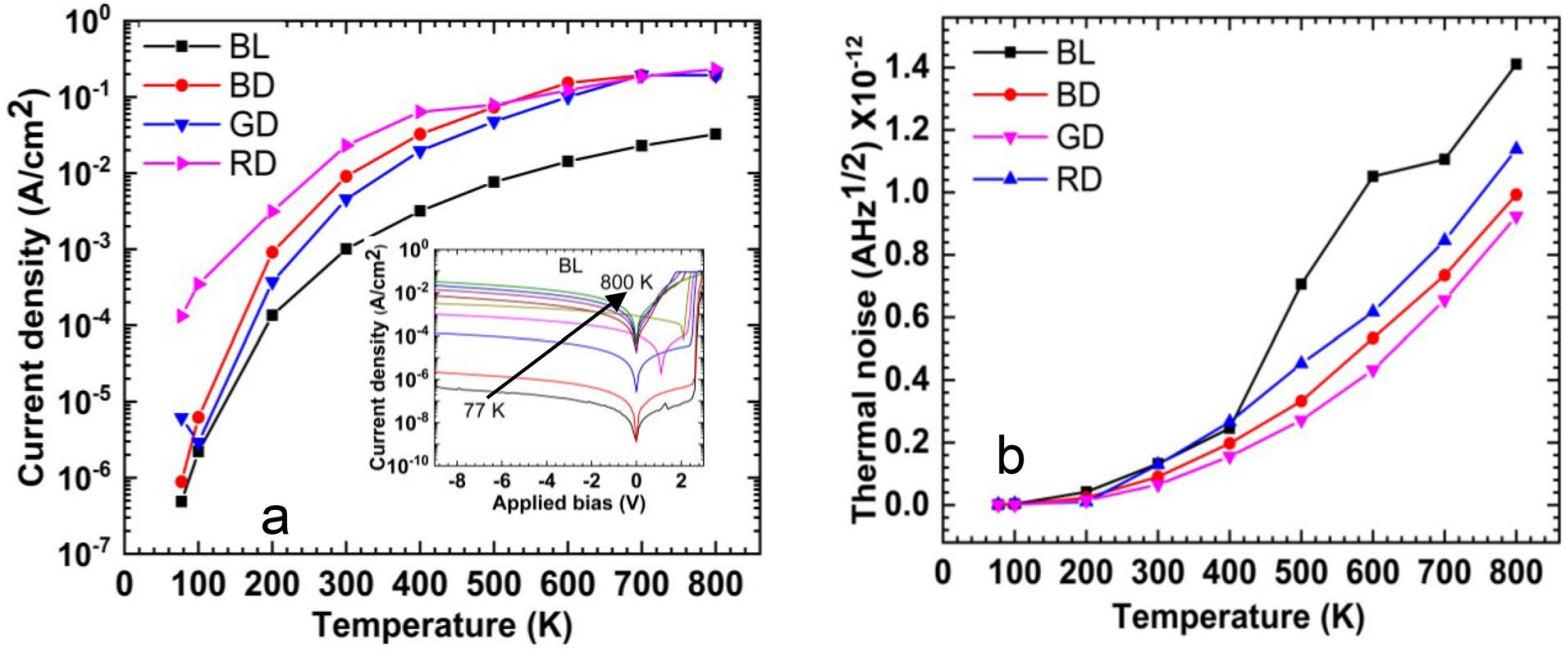
(**a**) Temperature-dependent leakage current density of blue for lighting and display, green for display, and red for display at − 9 V bias voltage (except red for display at − 7 V). Inset: Typical temperature-dependent I–V characteristics of blue for lighting. (**b**) Temperature-dependent thermal noise of devices.

The dark I–V characteristics of BL, BD, GD, and RD are studies, and they show similar behaviour with increasing the temperature. The typical temperature dependence dark I–V characteristics of BL as an inset in Fig. 3a. The leakage currents originate from surface recombination and bulk leakage. Leakage mechanisms consist of thermally excited carriers, generation-recombination in the depletion region, and tunnelling. Dark current generally increases with both applied bias voltage and with temperature. Moreover, the dark current flows through the same path as the photocurrent, so it will also have a shot noise term correlated with it. Since photodetectors contain parallel resistances, which are junction leakage resistances, there will be thermal noise present in the dissipative resistances of the photodetectors. As the temperature increases, the thermally excited carriers increase, leading to the rise of thermal noise. At high temperatures, the dark currents become a significant factor in evaluating the performance of devices, so it is necessary to understand the behaviour of the dark currents as temperature increases.

Figure 3a shows the temperature dependence leakage current density of the samples BL, BD, GD, and at − 9 V bias voltages (except for the RD, which is at − 7 V bias). BL shows the lowest leakage current density, while RD demonstrates the highest leakage current density as the temperature increases from 77 to 800 K. The leakage current is a function of both temperature and applied bias, implying a combination of tunnelling and thermally generated currents. The leakage current density of devices is increased by around four orders of magnitude by increasing the temperature from 77 to 800 K. It is also observed that a rapid increase (two orders of magnitude) in the leakage current density of the InGaN-based samples when the temperature is increased from 100 to 200 K. This rapid increase in the leakage current is due to the thermal ionization of carriers from deep traps and trap assisted tunnelling process.

The temperature-dependent thermal noises of the devices are plotted in Fig. 3b. GD exhibits the lowest thermal noise, while BL shows the highest thermal noise. Both Samples BD and GD reveal an increase in thermal noise by three orders of magnitude, while BL and RD show a rise in the thermal noise as the temperature increases from 77 to 800 K.

**Figure 4:**
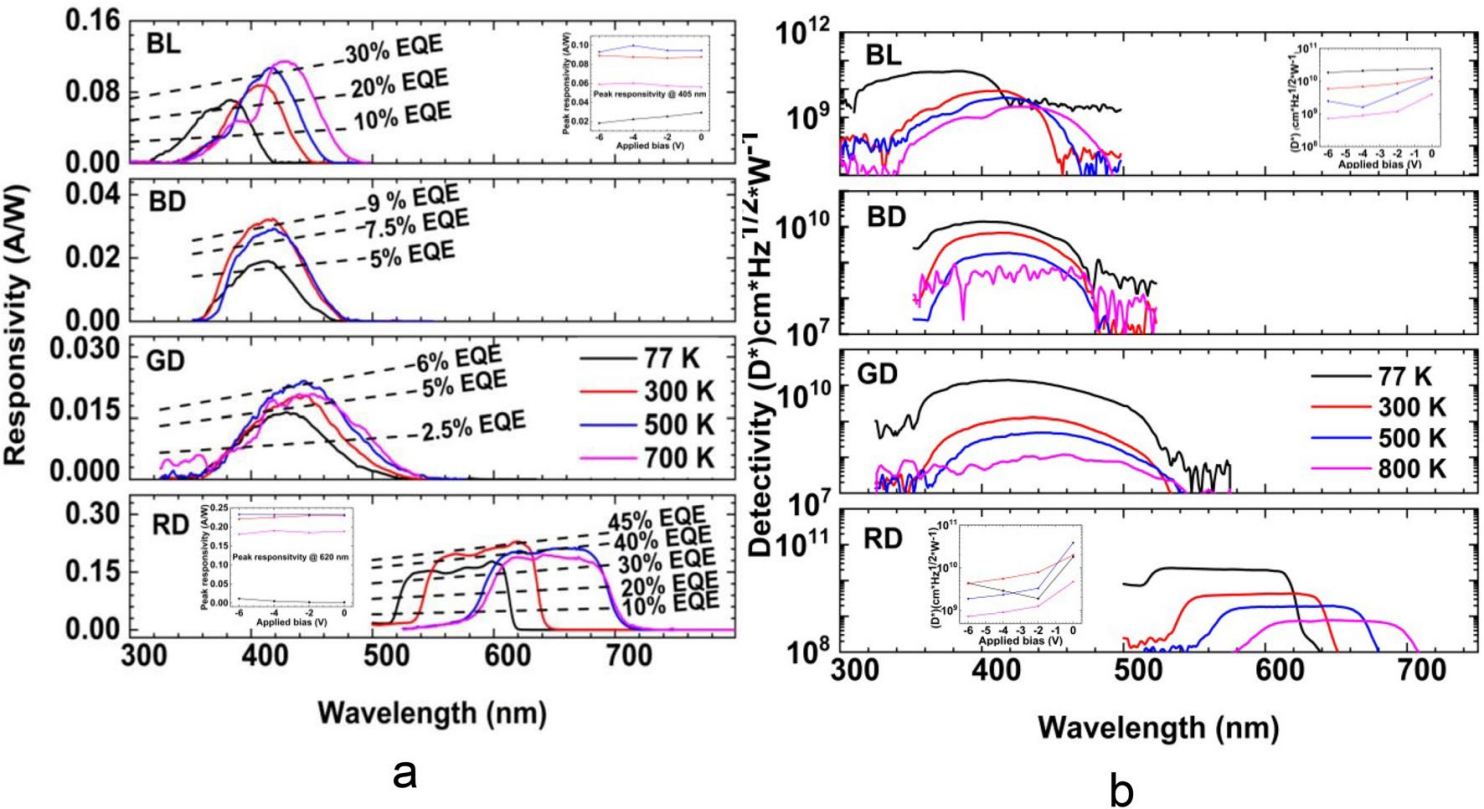
(**a**, **b**) Temperature-dependent responsivity and detectivity, respectively, of blue for lighting and display, green for display, and red for display at zero bias. Insets: Responsivity and detectivity as a function of different biases.

Figure 4a the temperature-dependent responsivity of all samples is plotted at 77 K (lowest temperature), 300 K (room temperature), 500 K (targeted operating temperature for the optocoupler), 800 K (highest temperature). Higher spectral response is noted from BL and RD. As the temperature increases, a redshift in the spectral response is more pronounced in BL and RD. The room temperature (RT) peak responsivity is 405, 418, 443, and 620 nm for BL, BD, GD, and RD, respectively. The peak position for sample BL changes from 383 to 428 nm by increasing the temperature from 77 to 800 K. The responsivity (@ 405) increases from 0.029 to 0.097 A W^−1^ by elevating the temperature from 77 to 600 K and decreases to 0.056 A W^−1^ at 800 K. The peak location for sample BD shifts from 402 to 418 nm by increasing the temperature 77–400 K and then moves to 394 nm at 600 K. The maximum responsivity from this device is 0.037 A W^−1^ at RT. The peak responsivity of sample GD changes from 430 to 450 nm by raising the temperature from 77 to 700 K and then shifts to 443 nm at 800 K. The peak responsivity (@ 443 nm) increases from 0.016 to 0.026 A W^−1^ as the temperature is elevated from 77 to 400 K and then decreases to 0.017 A W^−1^ by raising the temperature to 800 K. The peak position of RD moves from 598 to 667 nm when the temperature increases from 77 to 700 K and then changes to 642 nm at 800 K. The peak responsivity (@ 620 nm) increases from 0.003 to 0.235 A W^−1^ by raising the temperature from 77 to 400 K and droops to 0.189 A W^−1^ at 800 K.

**Figure 5:**
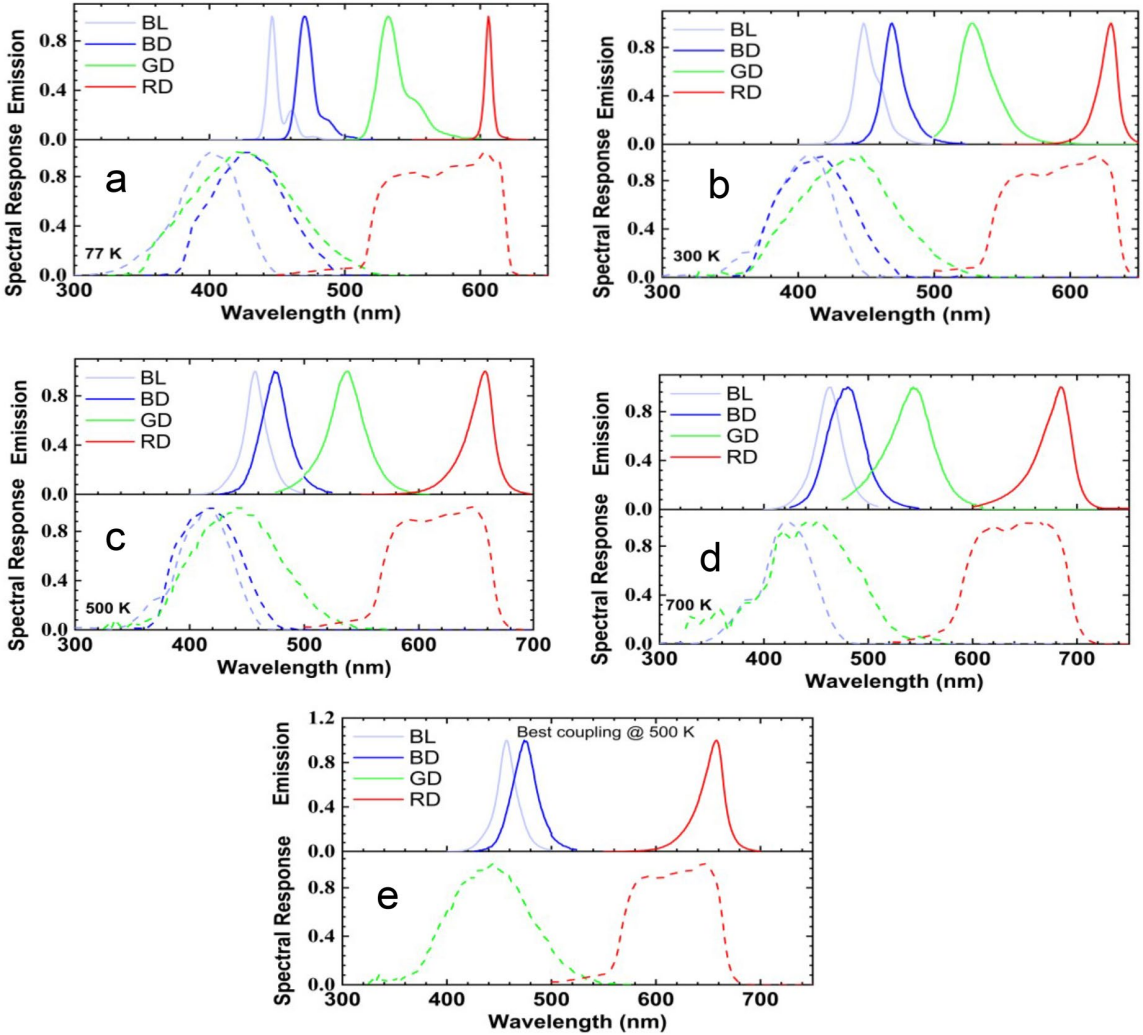
Four LEDs’ normalized emission spectra (solid) and spectral responses (dots) at (**a**) 77 K, (**b**) 300 K, (**c**) 500 K, (**d**) and 700 K. (**e**) Normalized emission and spectral response for selected LEDs for targeted high-temperature optocouplers.

The insets of Fig. 4a show the temperature-dependent peak responsivity at a different bias (0, 2, − 4, and − 6 V) for BL and RD. The peak responsivity of BL, BD, and GD show a slight increase with bias conditions, while RD indicates almost insensitivity to the bias conditions. The EQEs for all samples are shown in dotted lines in Fig. 5a. The highest and lowest EQE are observed from RD and GD, respectively.

Figure 4b shows the figure of merit, D*, of all LEDs, plotted at 77 K, 300 K, 500 K, 800 K using the 1 Hz equivalent noise bandwidth at the zero-bias condition. Overall, BD and GD show a lower signal to noise ratio compared with samples BL and RD. Furthermore, the peak D* of samples BD and GD are very close across all temperatures. The peak D* for BL decreases from 1.37 × 10^10^ to 3.94 × 10^9^ cm Hz^1/2^ W^−1^ by elevating the temperature from 77 to 800 K. However, RD shows an increase of the peak D* from 2.04 × 10^10^ to 5.21 × 10^10^ cm Hz^1/2^ W^−1^ by raising the temperature from 77 to 400 K. Then, increasing the temperature above 400 K leads to a decrease in peak D*. The peak D* is 7.66 × 10^9^ cm Hz^1/2^ W^−1^ at 800 K for RD. The insets of Fig. 4b show the D* of samples BL and RD with different biases. Both samples show high peak D* at zero bias conditions. Moreover, the same behaviour is observed for all LEDs. Table 1 lists the maximum values of responsivity and detectivity for various LEDs at 300, 500, and 800 K.

**Table 1:** Shows the peak $\mathfrak{R}$ and D* at 300, 500, and 800 K for the devices.

Sample	Temperature (K)	λ (nm)^a^	Peak $\mathfrak{R}$ (AW^−1^)	Bias (V)	Peak D* (cm Hz^1/2^ W^−1^) @ 0 bias
BL	300	411	0.093	− 6	1.37 × 10^10^
BD	300	411	0.035	− 6	6.84 × 10^9^
GD	300	437	0.038	− 6	5.69 × 10^9^
RD	300	620	0.23	− 2	1.99 × 10^10^
BL	500	418	0.11	− 2	1.26 × 10^10^
BD	500	423	0.031	− 4	1.87 × 10^9^
GD	500	443	0.034	− 6	1.89 × 10^9^
RD	500	646	0.25	0	4.07 × 10^10^
BL	800	428	0.16	− 2	3.94 × 10^9^
BD	800	430	0.020	0	9.34 × 10^8^ (@ 700 K)
GD	800	455	0.019	− 4	4.26 × 10^8^
RD	800	647	0.20	− 4	7.66 × 10^9^

^a^λ is the peak wavelength.

The normalized emissions and spectral responses of the LEDs at 77, 300, 500, and 700 K are plotted together in Fig. 5 to determine the best match of the LEDs and PDs for the optocouplers. It can be observed that the spectral response of the LED shifts towards shorter wavelengths (higher energy photons) as compared with its emission spectra as the LED cannot detect photons of lower energy than its bandgap. Table 2 shows the peak emission and spectral response wavelengths of the LEDs at 77, 300, 500, and 77 K. Overall, the perfect overlap is by employing samples RD-RD (LED-PD).

**Table 2:** Shows the peak emissions and spectral response at 77, 300, 500, and 800 K for the devices.

LED	Peak emission wavelength (nm)				Peak spectral response wavelength (nm)			
	77 K	300 K	500 K	700 K	77 K	300 K	500 K	700 K
BL	446	448	457	464	402	405	418	420
BD	470	469	474	480	402	418	418	420
GD	530	529	539	544	420	437	443	450
RD	606	630	658	684	605	620	646	667

The second option is by integrating samples BL-GD. Moreover, there is a possibility for coupling is by using BD-GD. Fig. 5a, b show the potential for coupling at 77 and 300 K when GD-RD are integrated. Fig. 5c shows the best candidates for the LEDs and PDs to fabricate HT optocouplers (i.e., operating at 500 K). Typically, the selection is based on the following parameters: (1) the wavelength; (2) maximize the signal to noise ratio (D*); (3) the forward current of the LEDs low as possible; (4) long-term reliability of the LEDs (degradation of LEDs due to operating time is acceptable).

## Discussions

**Figure 6:**
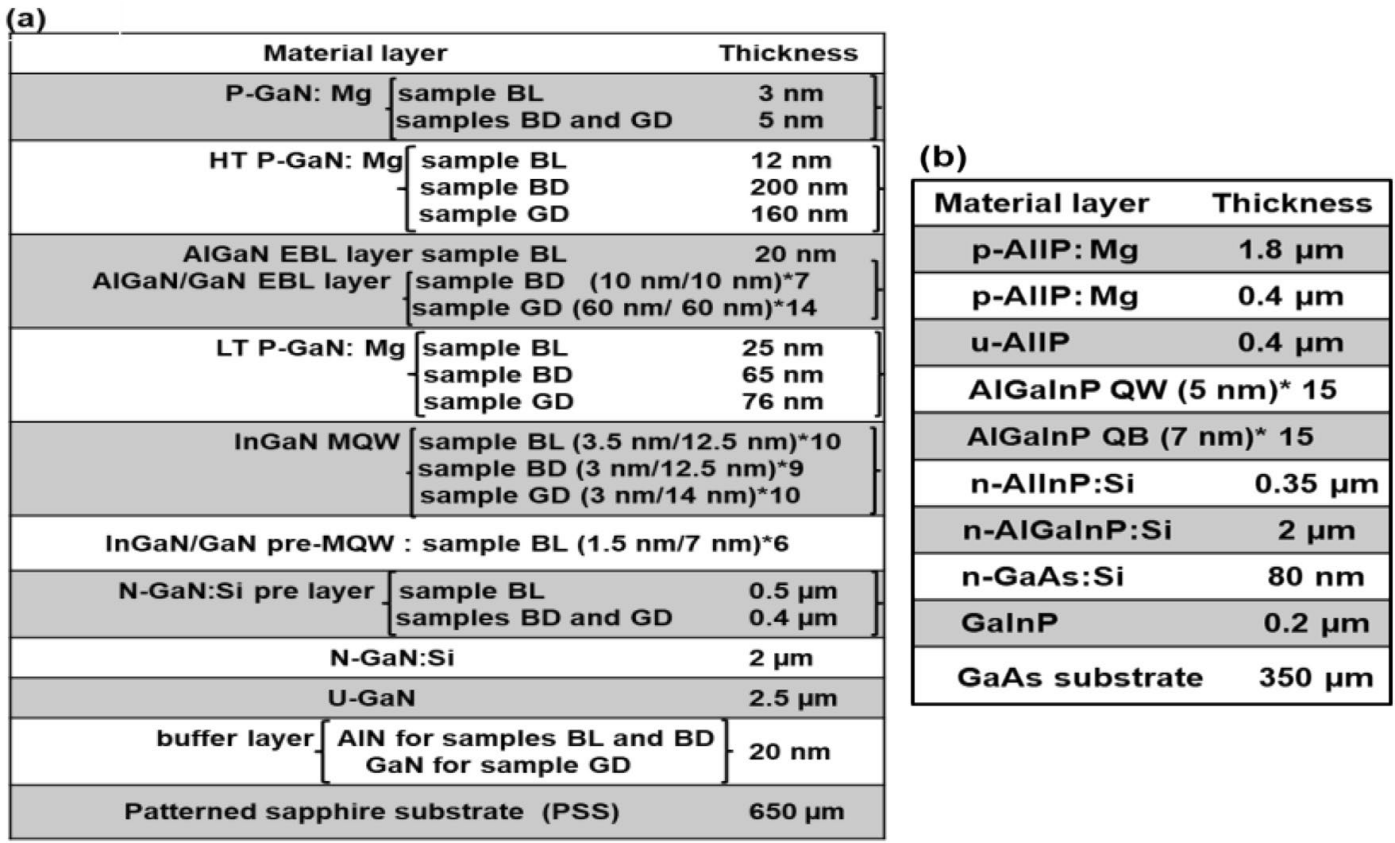
(**a**) A schematic diagram of the InGaN/GaN MQW LED epitaxial structure: BL, BD, and GD. (**b**) A schematic diagram of RD MQW LED epitaxial structure.

Based on the results from the bandwidth and responsivity of LEDs, it is observed that not all LEDs can serve as PDs. Commonly, the most extended emitted wavelength corresponds to approximately the highest responsivity of the LEDs. This behaviour agrees well with our results with one exception, which is BL. A previous study on AlGaN MQW PDs shows that the responsivity of PDs increases by increasing the number of wells and thickness of wells along with decreasing the width of the barriers^25^. First, as the number of wells increases, the effective absorption and efficiency increase, consequently increasing the responsivity. Second, when the thickness of wells increases, the transition energy between the electron and hole decreases resulting in increasing the responsivity. Third, the number of electron-hole pairs that contribute to photocurrent increases by reducing the thickness of the barriers. As the width of the barriers reduces, tunnelling through the barriers increases, leading to increase responsivity. The structures of the LEDs are shown in Fig. 6a. The main difference between samples BL, BD, and GD is the pre-quantum layer. The pre-quantum well layer adds more thickness to the active region. Sample A and C have the same number of wells, but they are different in the widths of the well and barriers as well as the pre-quantum well layer. The total thicknesses of the barriers are 44 and 30 nm for samples BL and GD, so the difference is 14 nm. On the other hand, the overall widths of the wells are 167 and 140 nm for samples BL and GD, so the difference is 27 nm. Therefore, it is expected that the thickness wells have more impact than the thickness of barriers. As a result, sample BL higher responsivity than sample GD due to the higher thickness in their wells. Now, the differences between samples BD and GD are QWs number and width of wells. Samples BD and GD have 9 and 10 QWs, respectively. Also, the thickness well is 12.5 and 14 nm for samples BD and GD, respectively. Consequently, sample GD has higher responsivity than sample BD. In short, BL has the highest responsivity among the InGaN/GaN MQW LEDs.

The D* depends on the responsivity and the area of the device. Since BL and RD have the highest responsivity and largest devices, they have higher D* than BD and GD. Furthermore, samples BD and GD show similar D* as they have the same device size.

Although the general responsivity of PDs increases as the reverse voltage increase, some PDs show insensitivity to the reverse voltage depending on the diode type. On the other hand, D* decreases for all PDs by applying the reverse bias. A high detectivity at zero biased condition suggests a high rate of change of noise than that of responsivity when the applied bias increases. This behaviour indicates that the detectivity of the MQW structure is limited to the bias-induced internal noise.

## Conclusion

The work demonstrates the possibility of fabricating HT optocouplers with targeting operating temperature of 500 K. Four LEDs are studied based on their emissions and spectral responses. BL, BD, GD, and RD are used in this article. The LEDs show EL spectra up to 800 K with relatively high EQE. BD and GD show higher EQE and lower efficiency droop with temperatures as compared with BL and RD. Also, this work proves that LEDs can function as PDs. In contrast with the LEDs, BL and RD LEDs perform superior PDs than others. The measured responsivity varies from 0.031 to 0.25 A W^−1^ at 500 K, depending on the type of LED. The figure of merit, D*, changes from 1.87 × 10^9^ to 4.07 × 10^10^ cm Hz^1/2^ W^−1^ at 500 K depending on the device. The EQE in temperature ranges from 77 to 800 K changes from 5 to 47 %, depending on the PD type. Finally, the wavelength (matching) overlap between the LEDs and PDs for different temperatures is demonstrated. First, the first possible couplings (LED–PD) are (RD–RD) and (GD–RD) in temperature ranging 77–300 K. Above 300 K, the potential couplings are (RD–RD), (BL–GD), (BD–GD). Therefore, (RD–RD) confirms that it can be employed by crossing all temperatures due to the high overlap between its emission and spectral response. In short, the present results indicate that LEDs can function as LEDs and PDs and use them to fabricate HT optocouplers, which can be substituted with the bulky transforms in the high-density power modules.

## Methods

**Figure 7:**
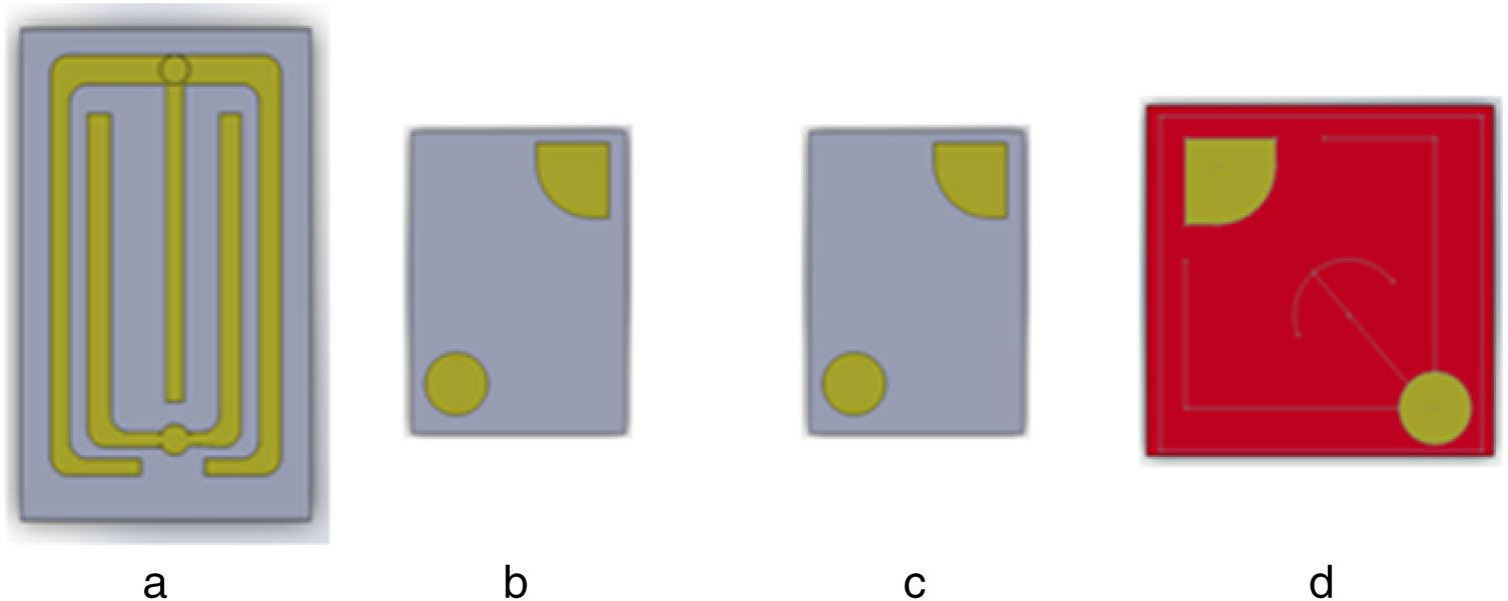
Images of LEDs. (**a**) Blue for lighting. (**b**) Blue for display. (**c**) Green for display. (**d**) Red for display.

The InGaN-based and AlGaInP-based multiple quantum wells (MQWs) LED structures are shown in Fig. 6a, b, respectively. Figure 6a shows the InGaN/GaN MQW LED structures for BL, BD, and GD LEDs. The room temperature peak wavelengths for BL, BD, and GD samples are 454, 472, and 523 nm, respectively. Figure 6b illustrates the Al_0.05_Ga_0.45_In_0.5_P/Al_0.4_Ga_0.1_In_0.5_P MQW LED structure for red for display (RD) with a room temperature peak wavelength of 630 nm. The detailed device structure has been discussed earlier^20–23,26^. The chip sizes are 800 × 1345 µm^2^ for BL LED, 191 × 270 µm^2^ for BD and GD LEDs, and 300 × 300 µm^2^ for RD LED. Figure 7a–d show LED images for blue for lighting and display, green for display, and red for display, respectively. These LEDs were supplied by HC SemiTek. The optical and electrical studies were conducted with a varied range of temperatures 77–800 K using a cryostat. The detail of the LEDs and photodiodes measurements and studied are explained previously^20–23,26^.

## References

[1] Sang L (2011). High-temperature ultraviolet detection based on InGaN Schottky photodiodes. Appl. Phys. Lett..

[2] De Vittorio M (2004). High temperature characterization of GaN-based photodetectors. Sens. Actuator. A- Phys..

[3] Hornberger J (2004). Silicon-carbide (Sic) semiconductor power electronics for extreme high-temperature environments. Aerosp. Conf. Proc..

[4] Ohadi, M. *et al*. Thermal Management of Harsh-Environment Electronics, Twentieth Annual IEEE Semiconductor Thermal Measurement and Management Symposium Proceedings, 231 (2004).

[5] Rosero J (2007). Moving towards a more electric aircraft. Aerosp. Electron. Syst. Mag..

[6] Fan H (2001). High-frequency transformer isolated bidirectional DC–DC converter modules with high efficiency over wide load range for 20 kVA solid-state transformer. IEEE Trans. Power Electron.

[7] Calfo R (2002). Generators for use in electric marine ship propulsion systems. IEEE Power Eng. Soc. Summer Meeting.

[8] Gerber M (2005). High density packaging of the passive components in an automotive DC/DC converter. IEEE Trans. Power Electron.

[9] Reatti A (2000). Low-cost high power-density electronic ballast for automotive HID lamp. IEEE Trans. Power Electron.

[10] Ilreike P (1994). An overview of high-temperature electronic device technologies and potential applications. Compon. Packag. Technol.

[11] Emadi A (2008). Power electronics and motor drives in electric, hybrid electric, and plug-in hybrid electric vehicles. IEEE Trans. Power Electron.

[12] Whitaker B (2014). A high-density, high-efficiency, isolated on-board vehicle battery charger utilizing silicon carbide power devices. IEEE Trans. Power Electron.

[13] Millan J (2014). A survey of wide bandgap power semiconductor devices. IEEE Trans. Power Electron.

[14] Neudeck P (2002). High-temperature electronics—A role for wide bandgap semiconductors. Proc. IEEE.

[15] Ray B (2005). High temperature operation of a DC–DC power converter utilizing SiC power devices. Appl. Power Elect. Co..

[16] Buttay, C. *et al*. State of the art of high temperature power electronics. Presented at the Microtherm, Lodz, Poland (2009).

[17] Lubura S (2014). Single-phase phase locked loop with DC offset and noise rejection for photovoltaic inverters. IEE Power Electron..

[18] Haghbin S (2013). Grid-connected integrated battery chargers in vehicle applications: Review and new solution. IEEE Trans. Ind. Electron.

[19] Sabbar A (2020). Systematic investigation of spontaneous emission quantum efficiency drop up to 800 K for future power electronics applications. IEEE J. Eme. Sel. Topics Power Electron..

[20] Sabbar A (2019). High temperature and power dependent photoluminescence analysis on commercial lighting and display LED materials for future power electronic modules. Sci. Rep..

[21] Sabbar A (2021). High-temperature spontaneous emission quantum efficiency analysis of different InGaN MQWs. IEEE J. Eme. Sel. Topics Power Electron..

[22] Madhusoodhanan S (2020). High-temperature analysis of GaN based blue LEDs for future power electronic applications. IEEE J. Eme. Sel. Topics Power Electron.

[23] Madhusoodhanan S (2020). High-temperature optical characterization of GaN-based light-emitting diodes for future power electronic modules. Phys. Status Solidi A..

[24] Oh C (2019). Current- and temperature-dependent efficiency droops in InGaN-based blue and AlGaInP-based red light-emitting diodes. Jpn. J. Appl. Phys..

[25] Rostami A (2014). High-responsivity AlGaN-GaN multi-quantum well UV photodetector. Int. J. Numer. Model Electron Netw. Devices Fields.

[26] Madhusoodhanan S (2021). High-temperature analysis of GaN-based MQW photodetector for optical galvanic isolations in high-density integrated power modules. IEEE J. Eme. Sel. Topics Power Electron.

